# Efficacy of repetitive transcranial magnetic stimulation combined with cognitive behavioral therapy for depression: a systematic review and meta-analysis

**DOI:** 10.3389/fpsyg.2026.1867678

**Published:** 2026-07-14

**Authors:** Qinxin Liu, Yihan Wang, Mengyuan Qi, Linhui Li, Hang Zhang

**Affiliations:** Department of Psychology, Anhui University of Chinese Medicine, Hefei, China

**Keywords:** cognitive behavioral therapy, combined treatment, depression, meta-analysis, repetitive transcranial magnetic stimulation, systematic review

## Abstract

**Background:**

Repetitive transcranial magnetic stimulation (rTMS) and cognitive behavioral therapy (CBT) are established treatments for depression. However, the incremental benefit of combining these approaches remains uncertain, partly because prior reviews have pooled studies with clinically heterogeneous comparator conditions. This study systematically reviewed the efficacy of rTMS combined with CBT for depression and conducted a meta-analysis restricted to trials using rTMS-based control conditions.

**Methods:**

We systematically searched Web of Science, PubMed, Embase, EBSCOhost, China National Knowledge Infrastructure (CNKI), Wanfang Data, VIP Database, and PsyArXiv from inception to 1 December 2025. Randomized controlled trials enrolling patients with depression and evaluating rTMS combined with CBT-spectrum interventions were eligible. Comparator conditions were stratified into rTMS controls, medication-based CBT controls, and medication controls. All eligible studies were included in the qualitative synthesis, whereas the quantitative synthesis was limited to studies comparing the combined intervention with rTMS controls. Standardized mean differences (Hedges’ g) were estimated using a random-effects model.

**Results:**

Ten randomized controlled trials involving 1,320 participants met the inclusion criteria. Qualitative synthesis suggested that the combined intervention may have advantages over both medication-based CBT controls and medication controls. Seven trials comparing the combined intervention with rTMS controls were included in the meta-analysis. The pooled analysis showed a small-to-moderate benefit of the combined intervention over rTMS control for reducing depressive symptoms (Hedges’ *g* = 0.38, 95% CI 0.13 to 0.63, *p* = 0.003), with moderate heterogeneity (*I*^2^ = 57.8%). Leave-one-out sensitivity analysis and cumulative meta-analysis indicated that the pooled estimate was stable. Egger’s test did not detect significant publication bias (*p* = 0.3914).

**Conclusion:**

Repetitive transcranial magnetic stimulation combined with CBT appears to provide additional benefit for the treatment of depression. Meta-analytic evidence supports greater symptom improvement relative to rTMS controls, and qualitative evidence suggests possible advantages over medication-based CBT controls and medication controls. These findings provide systematic support for the clinical potential of combining neuromodulation with psychotherapy while highlighting the need for larger, higher-quality trials with standardized protocols and longer follow-up.

**Systematic review registration:**

https://www.crd.york.ac.uk/PROSPERO/view/CRD420251267093, PROSPERO: CRD420251267093.

## 1 Introduction

Depression is among the leading mental disorders worldwide, affecting more than 264 million people (Cui et al., 2024) Although pharmacotherapy remains a first-line treatment for depression (Kern et al., 2020), approximately 10–30% of patients develop treatment resistance, with symptoms failing to improve despite adequate treatment with multiple antidepressants. Moreover, many patients experience adverse effects, including emotional blunting and weight gain (Cartwright et al., 2016; Johnco et al., 2024; Kennedy et al., 2002). These limitations have prompted increasing interest in alternative psychological and physical interventions.

Transcranial magnetic stimulation (TMS) is a noninvasive brain stimulation (NIBS) technique that delivers magnetic pulses over cortical regions, generating descending corticospinal impulses, activating neurons, and eliciting motor evoked potentials (MEPs). Repetitive transcranial magnetic stimulation (rTMS), in contrast to single-pulse TMS, delivers trains of pulses that can modulate cortical excitability beyond the stimulation period (Lefaucheur et al., 2020). In 2008, the U. S. Food and Drug Administration (FDA) approved rTMS for the clinical treatment of depression. A substantial body of evidence indicates that its efficacy depends on stimulation frequency, duration, and target site. Low-frequency stimulation (≤1 Hz) is generally thought to suppress local cortical activity; when applied to the right dorsolateral prefrontal cortex (R-DLPFC), it may help regulate abnormal lateralized activation within emotional networks. By contrast, high-frequency stimulation (≥5 Hz) tends to increase cortical excitability. The most commonly used protocol for depression is high-frequency stimulation over the left dorsolateral prefrontal cortex (L-DLPFC), a region that often shows reduced functional activity in depressed patients; increasing its excitability may help alleviate depressive symptoms (Klomjai et al., 2015). Nevertheless, the therapeutic effects of rTMS are shaped by multiple stimulation parameters. For example, stimulation intensities below or above threshold may reverse expected cortical excitability effects, contributing to between-study variability and ongoing controversy (Modugno et al., 2001). Against this background, combined biological and psychological treatment strategies have attracted growing interest, and pairing NIBS with psychotherapy, particularly CBT, has emerged as a promising direction.

Cognitive behavioral therapy (CBT) is one of the most widely used and best-supported psychotherapies. Since its development in the 1960s, it has continued to evolve and has repeatedly demonstrated efficacy for depression (Norcross et al., 2006). CBT emphasizes behavioral activation and cognitive restructuring, helping individuals adapt more effectively to specific situations, modify maladaptive thought patterns, and thereby reduce depressive symptoms (Ricou et al., 2019; Li et al., 2018) The National Institute for Health and Care Excellence (NICE) strongly recommends CBT as a first-line psychological intervention for mild, moderate, and severe depression. Its flexibility across face-to-face, online, and blended formats further improves accessibility and scalability across diverse populations. However, CBT also has limitations, including a relatively slow onset, high relapse rates in chronic depression after short-term treatment, and limited durability of benefit in severe or treatment-resistant cases (Tatti et al., 2022).

Accordingly, an increasing number of studies have combined rTMS with CBT. These interventions appear mechanistically complementary and may offset limitations inherent in each approach when used alone. By using neuromodulation to facilitate cognitive restructuring and behavioral activation, this combined biological-psychological strategy may improve overall therapeutic outcomes in depression.

In recent years, several studies have begun to evaluate the clinical application of rTMS combined with CBT for depression. Preliminary findings suggest that rTMS may enhance the effects of CBT (Miklowitz et al., 2021). More specifically, rTMS may induce neuroplastic changes that create a more favorable cognitive and emotional state for CBT delivery, thereby strengthening the effects of psychotherapy. Neuroplasticity has been regarded as a key therapeutic target underlying improvements in clinical symptoms, cognition, and functioning across a range of neuropsychiatric disorders (Wilkinson et al., 2019; Estela-Zape et al., 2025). For example, Xu et al. (2023), in a systematic review spanning 12 psychiatric conditions, reported that rTMS combined with psychological intervention improved overall clinical outcomes, suggesting transdiagnostic potential; however, that review included highly heterogeneous populations and did not include patients with depression (Xu et al., 2023). Within the depression literature, Khanna et al. (2026), in a scoping review of noninvasive brain stimulation combined with CBT, identified only one randomized controlled trial of rTMS combined with CBT, with most of the remaining evidence consisting of case reports or small open-label studies. The evidence base was therefore limited in methodological rigor and insufficient to exclude placebo effects, nonspecific treatment-contact effects, or to directly test potential synergy between rTMS and CBT (Khanna et al., 2026). To date, no systematic quantitative synthesis has specifically evaluated the efficacy of this combination for depression.

Accordingly, the present study conducted a systematic review and meta-analysis to evaluate the efficacy of rTMS combined with CBT for depression. Through a comprehensive literature search, we identified randomized controlled trials comparing the combined intervention with rTMS controls, medication-based CBT controls, or medication controls. Quantitative synthesis was restricted to studies using rTMS controls, for which both the number of eligible studies and the clinical comparability of control conditions were sufficient. Unlike previous reviews that pooled studies with heterogeneous comparator conditions, we stratified the evidence by comparator type and limited the meta-analysis to clinically comparable rTMS control trials. This approach was intended to reduce comparator-driven clinical heterogeneity, improve the interpretability of effect estimates, and more clearly define the added value of the combined strategy relative to different single-treatment approaches. We hypothesized that rTMS combined with CBT-spectrum interventions would produce greater improvement in depressive symptoms than rTMS control. Because comparisons with medication-based CBT controls and medication controls involved greater clinical heterogeneity and fewer comparable studies, these comparisons were examined qualitatively.

## 2 Methods

To maximize methodological rigor and reproducibility, this study was conducted in accordance with the Preferred Reporting Items for Systematic Reviews and Meta-Analyses (PRISMA) statement (Page et al., 2021) and was prospectively registered in PROSPERO (registration number: CRD420251267093).

### 2.1 Literature search

We conducted a comprehensive search of English- and Chinese-language databases. English-language studies were searched in Web of Science, PubMed, Embase, and EBSCOhost, whereas Chinese-language studies were searched in China National Knowledge Infrastructure (CNKI), Wanfang Data, and VIP Database. The search strategy in Web of Science was as follows:(TS = (“transcranial magnetic stimulat*” OR TMS OR rTMS OR “repetitive TMS” OR “brain stimulat*”))AND(TS = (“cognitive behavioral therap*” OR CBT OR “cognitive therap*” OR “behavioral activation”))AND(TS = (depress* OR “major depressive disorder” OR MDD OR “treatment resistant depress*”)).

The search covered the period from database inception to 1 December 2025. All retrieved records were imported into Zotero for record management, deduplication, and screening. To improve reproducibility, the full database-specific search strategies are provided in Supplementary Material 1. PsyArXiv^1^ was searched as a supplementary source to identify potentially relevant preprints and reduce publication bias. Records from PsyArXiv were screened using the same eligibility criteria as peer-reviewed publications. No PsyArXiv record met the inclusion criteria; therefore, no PsyArXiv preprint was included in either the qualitative synthesis or the quantitative meta-analysis. The final review included nine peer-reviewed articles and one registered clinical trial report. The clinical trial report was retained because it met the prespecified eligibility criteria, but its non-peer-reviewed status was considered when interpreting the evidence.

### 2.2 Eligibility criteria and study selection

Eligibility criteria were defined to align with the objectives of the meta-analysis.

Inclusion criteria: (1) participants met internationally recognized diagnostic criteria for depressive disorders, including DSM-5, ICD-10/ICD-11, or CCMD-3, with no restrictions on age, sex, or illness duration; (2) the intervention group received rTMS combined with CBT; (3) rTMS protocols included conventional high-frequency, low-frequency, or bilateral stimulation paradigms; (4) CBT-based interventions were explicitly grounded in cognitive and/or behavioral theoretical frameworks; in this review, standard individual or group CBT, internet- or therapist-guided CBT (iCBT), and mindfulness-based cognitive therapy (MBCT) were all classified within the broader CBT-spectrum intervention category (Gkintoni et al., 2025); (5) studies reported major pre- and post-intervention outcomes related to clinical symptoms (e.g., depressive symptom severity, functioning, or quality of life) or cognition using validated measures, with no restriction on a specific depression scale; and (6) the study design was a randomized controlled trial.

Exclusion criteria: (1) opinion papers, narrative reviews, editorials, non-randomized studies, case reports, case series, and systematic reviews; (2) studies using only rTMS or only CBT without combined intervention; (3) studies using mindfulness-only interventions without a cognitive-behavioral framework, unless explicitly defined as a CBT-derived therapy; (4) studies involving bipolar depression, psychotic depression, or primary neurodegenerative disorders; and (5) studies not published in Chinese or English.

**Figure 1:**
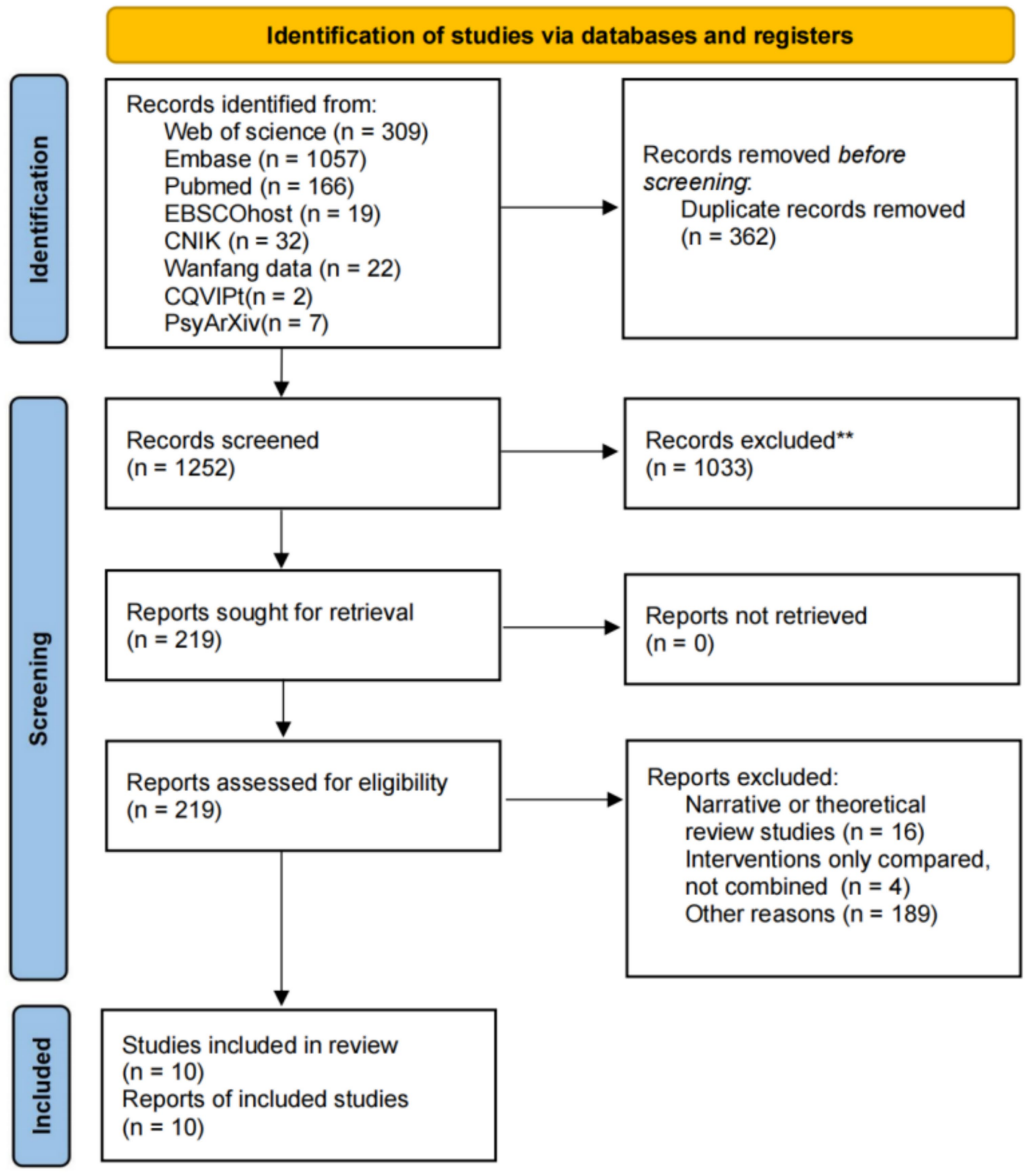
PRISMA flow diagram of the study selection process.

After duplicate removal, two reviewers independently screened titles and abstracts according to the eligibility criteria. Full texts of potentially eligible studies were then assessed. Disagreements were resolved through discussion until consensus was reached or, when necessary, through consultation with a third reviewer. The study-selection process is shown in Figure 1.

### 2.3 Data extraction and figure preparation

Two reviewers independently extracted data using a pilot-tested Excel form. Extracted information included: (1) study characteristics (author, year); (2) participant characteristics (sample size, age, sex); (3) intervention details (combined intervention and comparator type, CBT modality, session duration, session frequency, total number of sessions, treatment duration, and therapist involvement); (4) rTMS parameters (stimulation site, frequency, number of pulses, intensity); (5) outcome measures (depression scales and assessment time points); and (6) statistical data required for effect-size calculation (baseline and post-intervention scores); and (7) secondary outcomes where available, including adverse events, cognitive or functional outcomes, and dropout or discontinuation. When data were incomplete or unclear, corresponding authors were contacted by email for clarification. Any discrepancies in extraction were resolved through discussion. The extracted data were then organized into a standardized coding table for subsequent analysis.

### 2.4 Statistical analysis

Qualitative synthesis was used to summarize intervention characteristics and the distribution of outcome measures across the included studies. Quantitative analyses were conducted in R version 4.2.1 (R Core Team, 2022) and RStudio version 2022.12.0.353 (Posit Team, 2022) using the meta and metafor packages. Standardized mean differences (SMDs) with 95% confidence intervals (CIs) were calculated, and Hedges’ g was used to correct for small-sample bias. Heterogeneity was assessed using Cochran’s *Q* test and the *I*^2^ statistic; I^2^ > 50% was considered to indicate substantial heterogeneity (Huedo-Medina et al., 2006). A random-effects model was used to account for anticipated clinical and methodological heterogeneity.

Risk of bias was independently assessed by two reviewers using the Cochrane Risk of Bias tool for randomized trials (RoB 1) (Higgins and Green, 2011), with disagreements resolved by a third reviewer. The tool evaluates seven domains: (1) random sequence generation (selection bias); (2) allocation concealment (selection bias); (3) blinding of participants and personnel (performance bias); (4) blinding of outcome assessors (detection bias); (5) incomplete outcome data (attrition bias); (6) selective reporting (reporting bias); and (7) other bias. Each domain was rated as low, unclear, or high risk. Publication bias was assessed using funnel plots and Egger’s regression test (Egger et al., 1997).

## 3 Results

### 3.1 Characteristics of included studies

This systematic review and meta-analysis followed established procedures for literature screening and study inclusion. Ultimately, 10 randomized controlled trials were included, comprising 9 published articles and 1 clinical trial report (Rosenberg and University of California, Los Angeles, 2025). Details of the included studies are presented in Table 1. Of these studies, 7 were two-arm RCTs, 1 was a three-arm RCT, and 2 were four-arm RCTs. All used rigorous parallel-group designs, providing a structured evidence base for subsequent clinical application and future research.

**Table 1:** Characteristics of the included studies.

Study	Study design	Medication use	Sample Size (E/C)	Female (%)	Mean age ± SD	Experimental intervention	Control intervention	Integration	CBT modality	rTMS parameters					Depressive Outcome	Time	Baseline scale score	Post-intervention scale score
										Treatment sessions	Stimulation site	Frequency	Total Pulses	Intensity				
Wang and Du (2016)	Three-arm RCT	Yes	135(45/45/45)	EG: 77.8 CG1: 68.9 CG2: 73.3	EG: 21.93 ± 4.52 CG1: 22.77 ± 3.69 CG2: 21.35 ± 2.67	Drugs + rTMS + CBT	CG1: Drugs + rTMS CG2: Drugs	Concurrent	CBT	20	R-DLPFC	1 Hz	NF	100%RMT	HAMD-17	4 weeks	EG: 28.16 ± 3.95 CG1: 27.93 ± 5.01 CG2: 28.37 ± 4.86	EG: 17.92 ± 9.86 CG1: 18.30 ± 6.12 CG2: 19.44 ± 7.13
Wang et al. (2020)	Two-arm RCT	NF	86(43/43)	EG: 44.2 CG: 46.5	EG: 66.31 ± 2.54 CG: 66.41 ± 2.53	rTMS + CBT	rTMS	Concurrent	CBT	30	L-DLPFC	10 Hz	NF	90%RMT	HAMD-17	4 weeks	EG: 28.05 ± 4.67 CG: 27.98 ± 4.72	EG: 11.67 ± 5.86 CG: 13.25 ± 6.75
Qi et al. (2021)	Two-arm RCT	Yes	90(45/45)	EG: 55.6 CG: 62.2	EG: 47.21 ± 5.23 CG: 46.48 ± 4.92	Drugs + rTMS + MBCT	Drugs	Concurrent	MBCT	40	L-DLPFC	0.5 Hz	8,000	70%RMT	HAMD-17	8 weeks	EG: 27.42 ± 4.63 CG: 26.35 ± 4.76	EG: 11.03 ± 3.14 CG: 16.68 ± 4.25
Mao et al. (2022)	Two-arm RCT	NF	120(60/60)	EG:36.7 CG:31.7	EG: 34.08 ± 5.54 CG:33.50 ± 5.50	rTMS + CBT	rTMS	Concurrent	CBT	10	NF	<0.2 Hz	NF	500G	HAMD-17	2 weeks	EG: 21.09 ± 4.53 CG: 21.10 ± 4.55	EG: 4.85 ± 1.05 CG: 8.05 ± 1.12
Adu et al. (2023)	Two-arm RCT	NF	78(39/39)	EG:71.8 CG:56.4	EG: 39.24 ± 7.69 CG:37.64 ± 7.52	rTMS + iCBT	rTMS	Concurrent	iCBT	30	R-DLPFC L-DLPFC	1 Hz(R) 10 Hz(L)	1,200(R) 3,000(L)	120%RMT	HAMD-17	6 weeks	EG: 17.43 ± 4.79 CG: 15.73 ± 6.03	EG: 9.97 ± 7.03 CG: 8.89 ± 5.83
Li et al. (2023)	Four-arm RCT	Yes	296(75/74/73/74)	EG:59.0 CG1: 62.8 CG2: 61.5 CG3: 57.7	EG:66.80 ± 5.17 CG1:66.75 ± 5.26 CG2:67.21 ± 5.33 CG3:66.46 ± 5.08	Drugs + rTMS + CBT	CG1: Drugs + rTMS CG2: Drugs + CBT CG3: Drugs	Concurrent	CBT	12	L-DLPFC	1 Hz	1,500	100%RMT	HAMD-17	8 weeks	EG: 35.92 ± 4.62 CG1: 36.16 ± 4.32 CG2: 36.73 ± 4.14 CG3:35.97 ± 4.35	EG: 13.11 ± 2.64 CG1: 15.83 ± 3.57 CG2:16.69 ± 3.13 CG3:21.82 ± 3.76
Qiu et al. (2024)	Four-arm RCT	Yes	100(25/25/25/25)	EG:64.0 CG1:60.0 CG2:72.0 CG3:76.0	EG:15.76 ± 1.36 CG1:15.92 ± 1.82 CG2:15.20 ± 1.38 CG3:14.92 ± 1.32	Drugs + rTMS + CBT	CG: Drugs + rTMS CG2: Drugs + CBT CG3: Drugs	Concurrent	CBT	30	L-DLPFC	10 Hz	24,000	80%RMT	HAMD-24	4 weeks	EG:40.40 ± 10.45 CG1:37.92 ± 10.97 CG2:33.24 ± 7.80 CG3:35.40 ± 9.10	EG:15.00 ± 3.21 CG1:20.64 ± 7.89 CG2:24.76 ± 5.93 CG3:26.88 ± 6.89
Yao et al. (2024)	Two-arm RCT	Yes	290(145/145)	NF	EG:15.27 ± 2.69 CG:15.52 ± 2.48	Drugs + rTMS + CBT	Drugs	Concurrent	CBT	10	R-DLPFC	0.5 Hz	3,000	70%RMT	HAMD-24	8 weeks	EG: 21.78 ± 4.09 CG: 21.56 ± 3.86	EG: 8.76 ± 2.95 CG: 13.86 ± 3.17
Dalhuisen et al. (2024)	Two-arm RCT	Yes	89(48/41)	EG: 64.6 CG: 68.3	EG:44.8 ± 14.8 CG:42.3 ± 14.2	Drugs + rTMS + CBT	Drugs + CBT	Concurrent	CBT	25	L-DLPFC	10 Hz	3,000	120%RMT	HDRS-17	8 weeks	EG: 21.6 ± 4.1 CG: 21.2 ± 4.2	EG: 12.2 ± 7.4 CG: 14.6 ± 6.3
Rosenberg and University of California, Los Angeles (2025)	Two-arm RCT	NF	36(18/18)	EG: 33.3 CG: 55.6	EG:41.5 ± 0 CG: 41.5 ± 0	rTMS + CBT	rTMS	Concurrent	iCBT	NF	NF	NF	NF	NF	HAMD-17	6 weeks	EG: 20.44 ± 4.10 CG: 21.77 ± 4.60	EG: 11.33 ± 6.63 CG: 11.92 ± 8.80

The included studies were published between 2016 and 2025. The total sample comprised 1,320 participants, with individual study sample sizes ranging from 36 to 296. All participants were diagnosed with depressive disorders. Ages ranged from adolescence to older adulthood, with mean ages between 15 and 67 years. Women accounted for 33.3 to 77.8% of participants across studies, which is broadly consistent with the epidemiology of depression. Clinically, all studies focused on unipolar depression, most commonly of moderate to severe severity. During the intervention period, 6 studies involved concomitant antidepressant medication use, whereas 4 did not clearly report medication status.

The operational parameters of CBT-spectrum interventions varied considerably across studies and were often incompletely reported. Where available, session duration ranged from 30 to 60 min, treatment duration ranged from 2 to 8 weeks, and session frequency ranged from once to twice weekly. Therapist involvement ranged from fully guided face-to-face delivery to unguided self-help with minimal contact. Several studies did not report session duration, total number of sessions, or therapist qualifications. Detailed CBT operational parameters are provided in Supplementary Table S1.

All experimental groups received rTMS combined with a CBT-spectrum intervention, including standard CBT, MBCT, and iCBT, and all studies implemented the combined treatment concurrently. rTMS target sites were primarily the left or right dorsolateral prefrontal cortex. Stimulation frequencies ranged from 0.5 to 10 Hz. With the exception of Mao et al. (2022), which used a fixed intensity of 500 Gauss, stimulation intensity in the remaining studies was expressed as 70–120% of the resting motor threshold (%RMT). Notably, %RMT represents a relative, individualized intensity metric based on resting motor threshold, whereas Gauss (Gs) denotes an absolute physical measure of magnetic field strength. Treatment duration ranged from 2 to 8 weeks. Comparator conditions were classified into three categories: rTMS controls, medication-based CBT controls, and medication controls. All studies used Hamilton Depression Rating Scale variants (HAMD-17, HAMD-24, or HDRS-17) to assess depressive symptoms. Baseline scores were balanced across groups, indicating good comparability.

**Figure 2:**
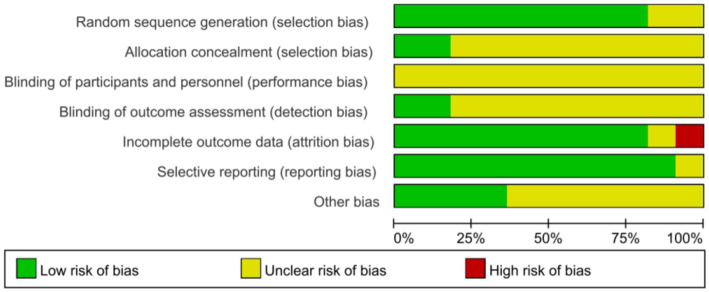
Risk-of-bias graph of the included studies.

**Figure 3:**
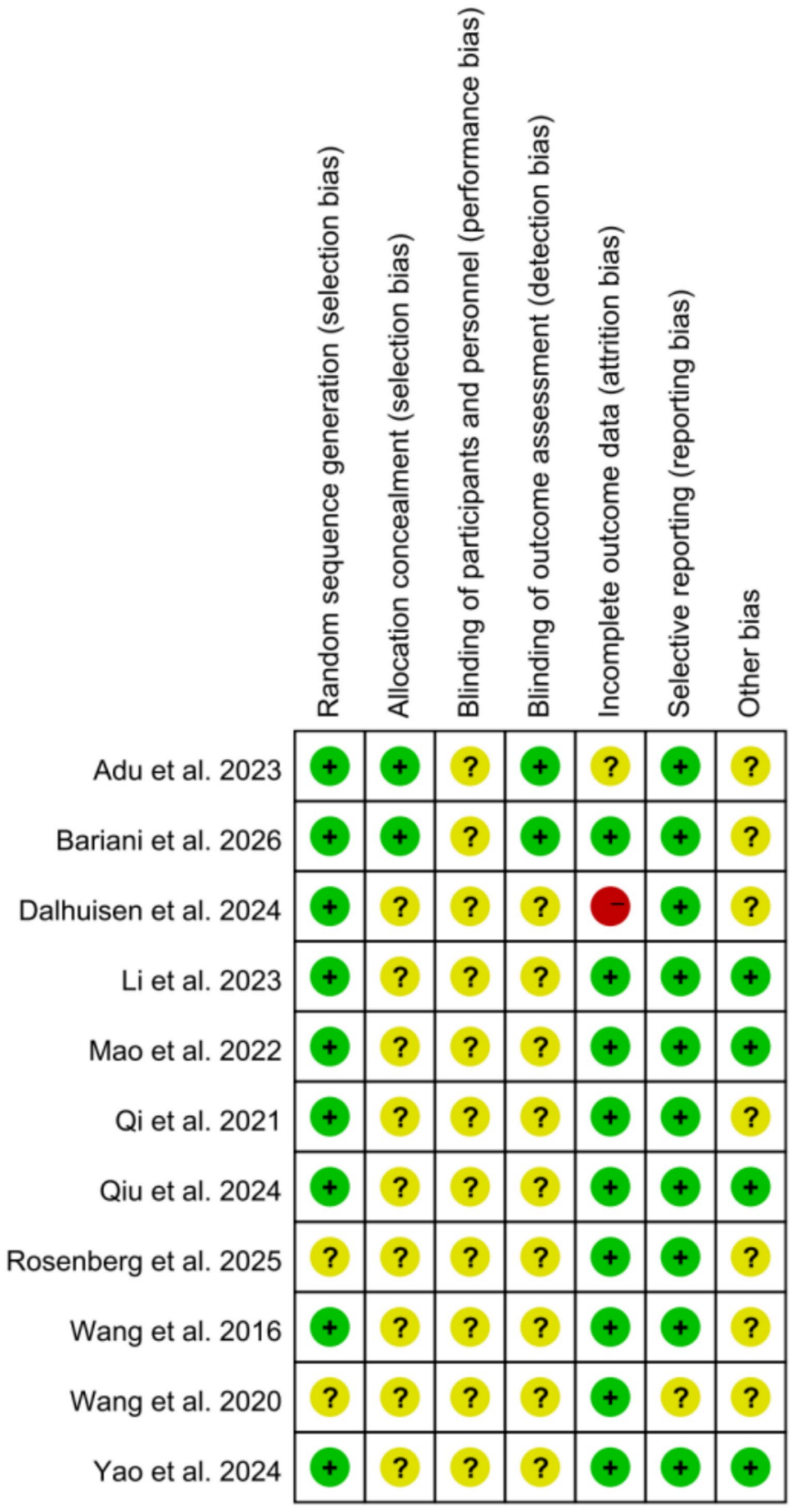
Risk-of-bias summary of the included studies.

Risk-of-bias assessment results are shown in Figures 2, 3. All studies were rated as low risk for random sequence generation. Allocation concealment was insufficiently described in many studies, resulting in some uncertainty. Because of the nature of the interventions, blinding of participants and personnel was not feasible in most studies; performance bias was therefore generally judged to be high. By contrast, most studies used blinded outcome assessment and were rated as low risk for detection bias. Incomplete outcome data and selective reporting were generally well controlled, with most studies receiving low-risk ratings. Blinding of participants and therapists was difficult in most included studies because rTMS produces perceptible scalp sensations and muscle twitching, whereas CBT involves direct therapist-patient interaction. This limitation is common in trials of combined physical and psychological interventions, but it may increase expectancy effects, nonspecific treatment-contact effects, and performance bias. As a result, the pooled effect may overestimate the specific benefit of the combined intervention. Although most studies used randomized allocation and blinded outcome assessment, the limited feasibility of participant and therapist blinding should be considered when interpreting the validity and magnitude of the observed effect. Overall, the included studies were of acceptable quality in terms of randomization, outcome assessment, and data completeness, although the pervasive risk of performance bias should be considered when interpreting the findings.

### 3.2 Narrative synthesis of main findings

Because several included studies used three-arm or four-arm designs, a single study could contribute to more than one comparison category. Pooling studies with different comparator conditions into a single analysis would have introduced substantial clinical heterogeneity and reduced the interpretability of relative efficacy against specific single-treatment approaches. For greater clinical clarity, studies were therefore grouped by comparator type and synthesized narratively as follows: (1) combined treatment versus rTMS control (7 studies), (2) combined treatment versus medication-based CBT control (3 studies), and (3) combined treatment versus medication control (5 studies).

#### 3.2.1 Combined treatment versus rTMS control

Seven studies compared combined treatment with rTMS control, and all used randomized controlled designs. Six were two-arm studies (85.7%), and one was a four-arm study (14.3%). Sample sizes ranged from 36 to 120 participants. The total sample size was 330 in the combined-treatment groups and 330 in the rTMS control groups. Participants ranged from adolescents to older adults.

Regarding treatment parameters, the L-DLPFC was the most common stimulation site (5 studies, 71.4%), followed by the R-DLPFC (2 studies, 28.6%). Stimulation frequencies included 1 Hz, 10 Hz, 0.5 Hz, and <0.2 Hz, and the number of treatment sessions ranged from 10 to 30. Traditional face-to-face CBT was the predominant psychotherapeutic modality (5 studies, 71.4%), whereas iCBT was used in two studies (28.6%). For depression outcomes, five studies reported greater post-treatment improvement in HAMD scores in the combined-treatment group than in the rTMS control group, including Mao et al. (2022), Wang and Du (2016), Wang et al. (2020), Li et al. (2023), and Qiu et al. (2024). For example, in Mao et al. (2022), the reduction in HAMD-17 scores was greater in the combined-treatment group than in the rTMS control group (post-treatment scores: 4.85 ± 1.05 vs. 8.05 ± 1.12). Similarly, Wang et al. (2020) reported more pronounced improvement in the combined-treatment group (11.67 ± 5.86 vs. 13.25 ± 6.75). However, Adu et al. (2023) and Rosenberg and University of California, Los Angeles (2025) did not find statistically significant between-group differences.

#### 3.2.2 Combined treatment versus medication-based CBT control

Three studies compared combined treatment with medication-based CBT control, and all were randomized controlled trials. Two were four-arm studies (66.7%), and one was a two-arm study (33.3%). Sample sizes ranged from 48 to 148 participants. The total sample size was 148 in the combined-treatment groups and 139 in the CBT control groups.

All three studies used L-DLPFC stimulation. Stimulation frequencies were 1 Hz or 10 Hz, and the number of treatment sessions was 12, 25, or 30. Traditional CBT was used as the psychotherapeutic component in all three studies.

For depression outcomes, all three studies suggested that the combined-treatment group may have achieved greater improvement in depression scores than the medication-based CBT control group. For example, in Li et al. (2023), the reduction in HAMD-17 scores was greater in the combined-treatment group than in the CBT control group (post-treatment scores: 13.11 ± 2.64 vs. 16.69 ± 3.13). Similar patterns were reported by Qiu et al. (2024) and Dalhuisen et al. (2024). Detailed parameters and score data are provided in Table 1.

#### 3.2.3 Combined treatment versus medication control

Five studies compared combined treatment with medication control, and all were randomized controlled trials. Two were two-arm studies (40.0%), one was a three-arm study (20.0%), and two were four-arm studies (40.0%). Sample sizes ranged from 50 to 290 participants. The total sample size was 335 in the combined-treatment groups and 334 in the medication control groups.

The L-DLPFC was the most common stimulation site (3 studies, 60.0%), followed by the R-DLPFC (2 studies, 40.0%). Stimulation frequencies included 1 Hz, 10 Hz, and 0.5 Hz, and the number of treatment sessions ranged from 10 to 40. CBT was the primary psychotherapeutic modality (4 studies, 80.0%), whereas MBCT was used in one study (20.0%).

For depression outcomes, all five studies suggested that the combined-treatment group may have achieved greater improvement in depression scores than the medication control group. For example, in Yao et al. (2024), HAMD-24 scores decreased from 21.78 ± 4.09 to 8.76 ± 2.95 in the combined-treatment group, compared with a decrease from 21.56 ± 3.86 to 13.86 ± 3.17 in the medication control group. Qi et al. (2021) and Li et al. (2023) reported similar patterns. Overall, the qualitative evidence suggests that combined treatment may offer some advantage in reducing depressive symptoms. Detailed study parameters and scores are presented in Table 1.

#### 3.2.4 Summary of narrative findings

Across the three comparator categories, combined treatment (rTMS + CBT/iCBT/MBCT) generally showed a trend toward better outcomes than each single-treatment approach. The largest evidence base was for combined treatment versus rTMS control (7 studies). With the exception of Adu et al. (2023) and Rosenberg and University of California, Los Angeles (2025), which found no statistically significant differences, the remaining five studies supported greater improvement in depressive symptoms with combined treatment relative to rTMS control. For comparisons with medication-based CBT control and medication control, the qualitative evidence also consistently suggested a potential advantage of combined treatment. Overall, the available qualitative evidence indicates that adding a CBT-spectrum intervention to rTMS, CBT, or medication may further enhance therapeutic benefit in depression. Because the comparison with rTMS control involved the largest number of eligible studies and the most clinically comparable question, this subgroup was selected for quantitative meta-analysis. Secondary outcomes were reported inconsistently across the included studies. Adverse events were described in only a minority of studies; where reported, they were generally mild, and no serious adverse events were documented. Cognitive outcomes were assessed in a small number of studies using heterogeneous instruments, including RBANS, memory tests, MoCA, TSCS, and PSP. Dropout and missing-data handling were also incompletely reported. These data were therefore summarized narratively, and detailed extracted information is provided in Supplementary Table S2.

### 3.3 Meta-analysis of combined treatment versus rTMS control

#### 3.3.1 Primary analysis

Because the included studies used different versions of the Hamilton Depression Rating Scale, standardized mean differences were used to minimize the influence of scale-version differences on effect-size estimation. The original studies reported pre- and post-intervention scores but did not provide pre–post correlation coefficients. Following recommendations from the Cochrane Handbook, we assumed a correlation coefficient of r = 0.5 to estimate standardized mean differences based on change scores.

**Figure 4:**
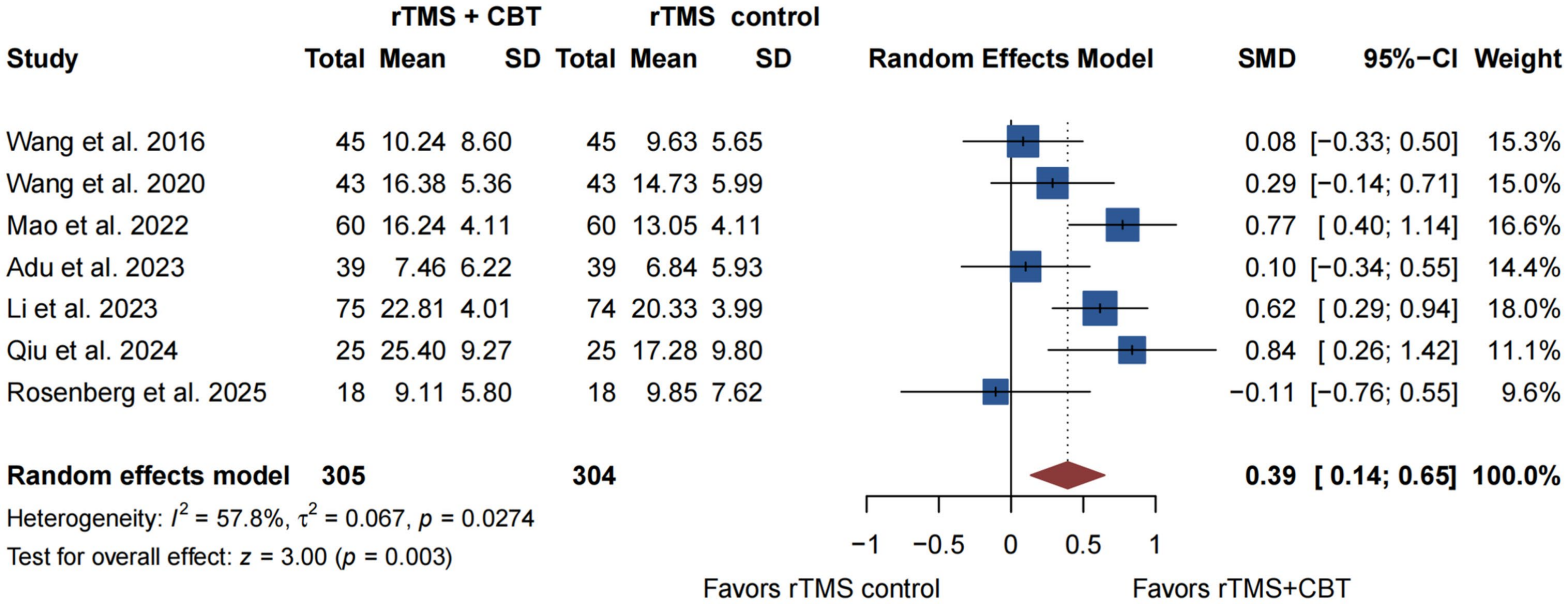
Forest plot of the meta-analysis comparing combined treatment versus rTMS control for depressive symptoms.

Seven randomized controlled trials were included. Heterogeneity testing indicated moderate between-study heterogeneity (*I*^2^ = 57.8%, *p* = 0.0274), so a random-effects model was used. The pooled effect showed that, relative to the rTMS control group, the combined-treatment group achieved significantly greater improvement in depressive symptoms, with a Hedges’ *g* of 0.38 (95% CI 0.13 to 0.63, *z* = 3.00, *p* = 0.003). The corresponding forest plot is shown in Figure 4. This effect falls within the small-to-moderate range (0.2 = small, 0.5 = moderate, 0.8 = large), suggesting that rTMS combined with a CBT-spectrum intervention is superior to rTMS control alone for improving depressive symptoms.

#### 3.3.2 Subgroup analysis

To examine potential sources of heterogeneity, we conducted exploratory subgroup analyses by CBT modality, rTMS frequency, and medication status. CBT delivery format appeared to be the most informative moderator. Face-to-face CBT combined with rTMS showed a significant benefit relative to rTMS control, whereas iCBT combined with rTMS did not. The between-subgroup difference was statistically significant, suggesting that treatment format may partly explain the moderate heterogeneity observed in the primary analysis. By contrast, subgroup analyses by rTMS frequency and medication status did not show significant between-subgroup differences. These analyses should be interpreted cautiously because the number of studies in each subgroup was small and statistical power was limited. MBCT was represented by one study and was included only in the qualitative synthesis of the medication-control comparison, not in the quantitative meta-analysis against rTMS control. Detailed subgroup results and forest plots are presented in the Supplementary Material 2 (Figures R1–R3).

#### 3.3.3 Publication bias

**Figure 5:**
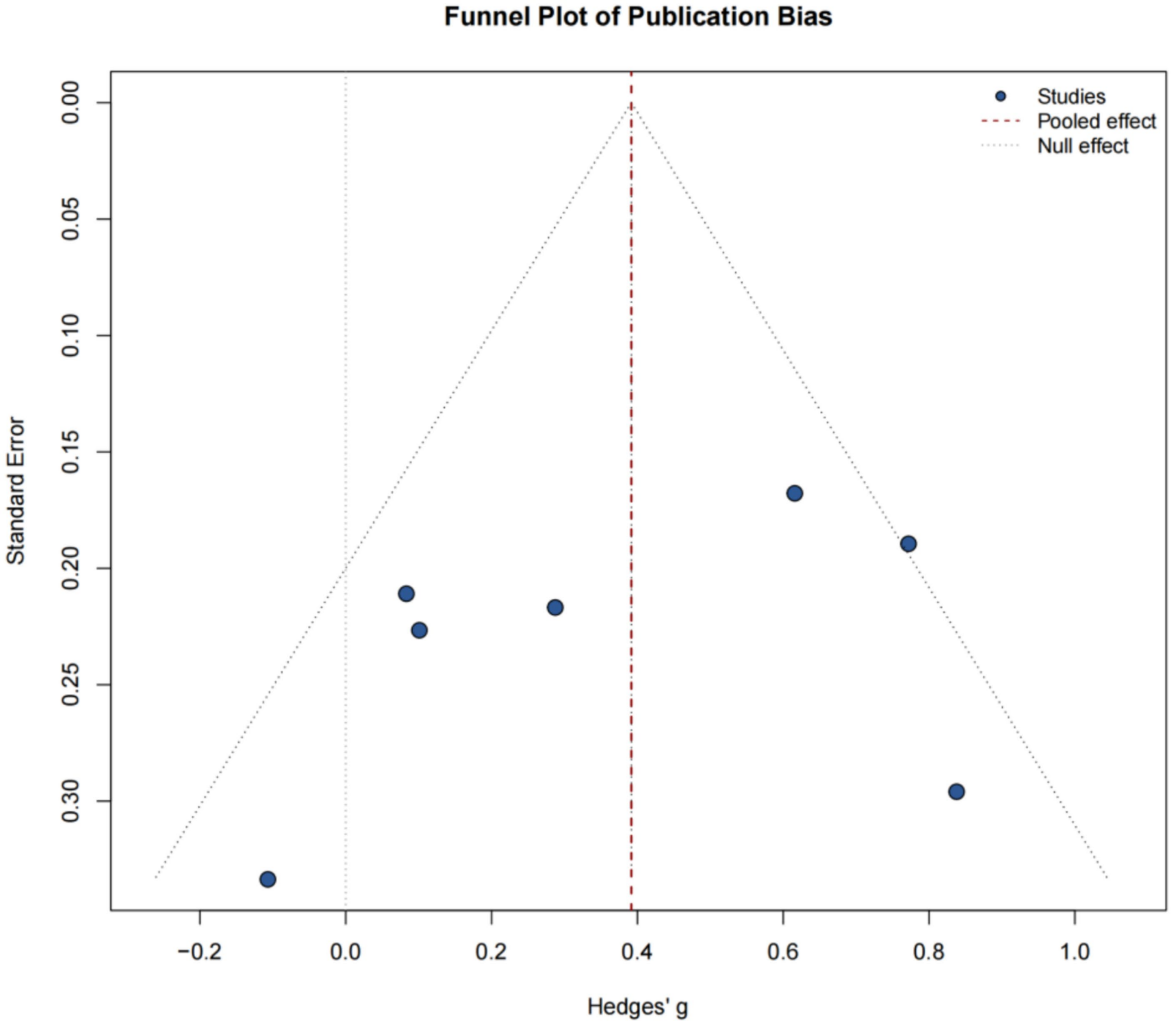
Funnel plot for publication bias in the meta-analysis comparing combined treatment versus rTMS control.

Publication bias was evaluated using a funnel plot and Egger’s linear regression test. The funnel plot (Figure 5) showed that study estimates were distributed approximately symmetrically around the pooled effect size, with no obvious asymmetry. Egger’s test yielded *t* = −0.94, df = 5, *p* = 0.3914, which was not statistically significant (*p* > 0.05). Taken together, the funnel plot and Egger’s test did not indicate significant publication bias, suggesting that the available evidence was relatively robust.

#### 3.3.4 Sensitivity analysis and cumulative meta-analysis

**Figure 6:**
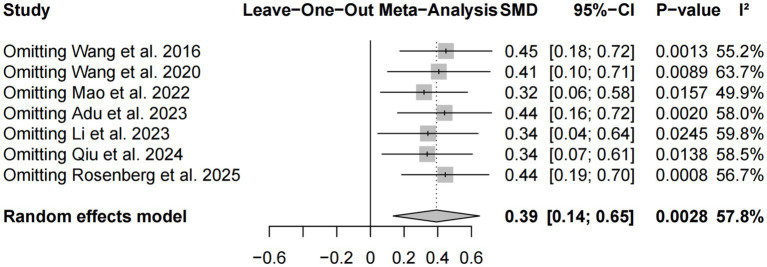
Leave-one-out sensitivity analysis of the meta-analysis comparing combined treatment versus rTMS control.

A leave-one-out sensitivity analysis was conducted to assess the influence of individual studies on the pooled effect size. As shown in Figure 6, omitting any single study yielded pooled Hedges’ g values ranging from 0.32 to 0.45. In all cases, the 95% confidence intervals remained above zero, indicating that the intervention effect remained statistically significant (*p* < 0.05 for all iterations). The *I*^2^ statistic remained within a moderate range (49.9–63.7%), suggesting that the observed heterogeneity was not driven by any single study.

Because pre-post correlations were not reported in the original studies, the primary analysis assumed *r* = 0.5. To examine the robustness of this assumption, we repeated the analysis using assumed correlations from 0.2 to 0.8. Across all assumed values, the pooled effect remained positive and statistically significant. The pooled Hedges’ *g* ranged from 0.34 to 0.50, and no confidence interval crossed zero. These results indicate that the estimated benefit of rTMS combined with CBT relative to rTMS control was robust to alternative assumptions regarding the pre-post correlation. The sensitivity analysis using alternative assumed pre-post correlations is presented in Supplementary Material 2 (Figure R4).

**Figure 7:**
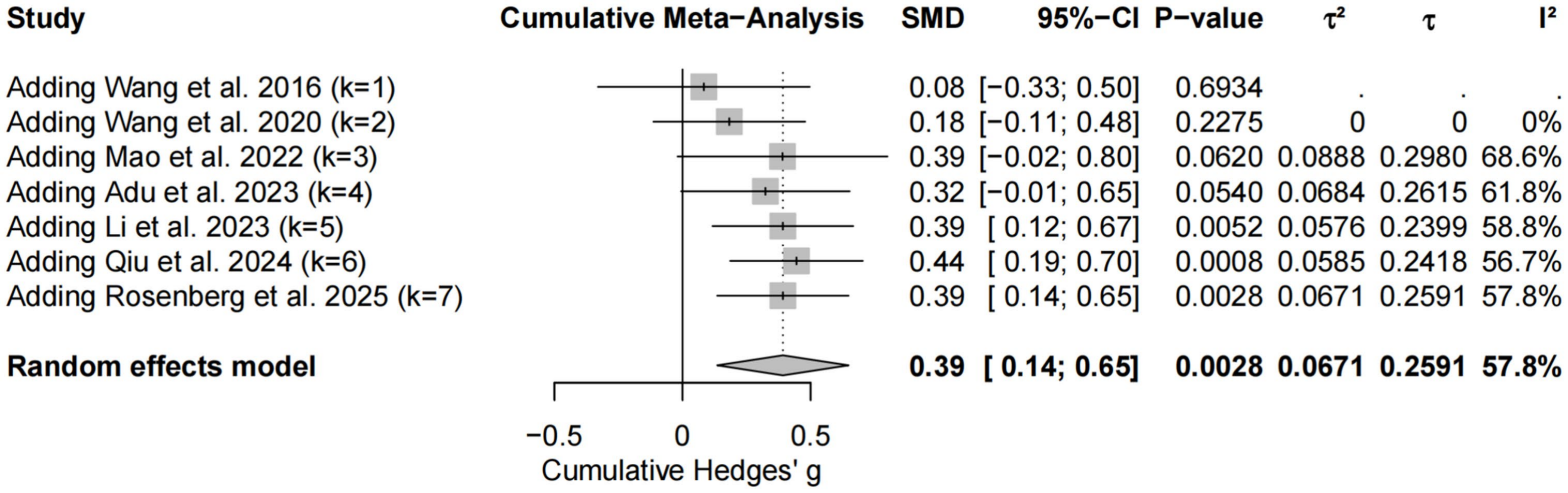
Forest plot of the cumulative meta-analysis by publication year comparing combined treatment versus rTMS control for depressive symptoms.

A cumulative meta-analysis by publication year (Figure 7) showed that when only a small number of early studies were included (*k* ≤ 4), the pooled effect size fluctuated considerably, the confidence intervals were wide and crossed zero, and the effect was not statistically significant. As the number of studies increased (*k* ≥ 5), the pooled effect stabilized at approximately 0.39–0.44, the 95% confidence intervals no longer crossed zero, and heterogeneity remained at a moderate level (*I*^2^ = 56.7–68.6%). These results indicate that, as the evidence base has accumulated, the estimated benefit of rTMS combined with CBT relative to rTMS control has become more stable. Nevertheless, given the limited number of included studies, this cumulative pattern still requires confirmation in larger, higher-quality trials.

## 4 Discussion

### 4.1 Main findings

To our knowledge, this study provides the first combined qualitative and quantitative evaluation of the efficacy of rTMS combined with CBT for depression. A total of 10 randomized controlled trials involving 1,320 participants were included. Because several studies used multi-arm designs, a single trial could contribute to more than one comparator category. To more accurately estimate the relative efficacy of combined treatment against specific single-treatment approaches, we stratified the synthesis by comparator type. The findings indicate that meta-analytic evidence supports greater improvement in depressive symptoms with combined treatment relative to rTMS control, whereas qualitative evidence suggests that combined treatment may also be advantageous relative to medication-based CBT control and medication control.

Notably, two studies (Adu et al., 2023; Rosenberg and University of California, Los Angeles, 2025) did not detect a significant incremental benefit of combined treatment over rTMS control. Closer examination showed that both studies used internet-based CBT (iCBT) as the psychotherapeutic component. iCBT is a digital extension of traditional CBT and is intended to address limitations in accessibility and scalability in routine clinical practice (Murray et al., 2009). Its major advantages include reduced dependence on therapist availability, broader access, and lower implementation costs. However, it also requires patients to complete treatment modules more independently and often provides less immediate therapist feedback than face-to-face CBT. Depending on the availability of therapist support, iCBT can be classified as guided or unguided. Previous research suggests that guided iCBT is more effective than unguided iCBT and that therapist support may improve patient motivation and treatment adherence (Cuijpers et al., 2015; Cuijpers et al., 2019). Adu et al. (2023) used an unguided iCBT program (MoodGYM), and the authors also noted that the platform, which was developed in Australia, may not have been optimally culturally adapted for the North American population studied. Rosenberg and University of California, Los Angeles (2025), by contrast, used a guided iCBT program (This Way Up) delivered through UCLA clinical services. However, this study was a clinical trial report rather than a peer-reviewed journal publication, involved a small sample (*n* = 36), and did not report key rTMS parameters such as stimulation site, frequency, or pulse number. The investigators contacted the authors for clarification but did not receive a response. These factors may have reduced statistical power and increased the risk of bias.

The meta-analysis comparing combined treatment with rTMS control yielded a small-to-moderate effect size (Hedges’ *g* = 0.38) with moderate heterogeneity (*I*^2^ = 57.8%). Compared with earlier exploratory syntheses (Khanna et al., 2026), heterogeneity was lower in the present analysis, suggesting that comparator stratification may have improved the coherence and interpretability of the pooled estimate. Some of the inconsistency reported in earlier syntheses may have reflected variation in comparator conditions rather than instability in the effect of the combined intervention itself. Sensitivity analysis and cumulative meta-analysis further indicated that the observed effect was not driven by any single study and became increasingly stable as evidence accumulated (Goldsworthy et al., 2021). Given the limited number of studies, we were unable to formally examine sources of heterogeneity through meta-regression. Nonetheless, plausible contributors to the residual heterogeneity include: (1) variation in treatment parameters, such as rTMS frequency (0.2–10 Hz), total pulse number (1200–24,000), and stimulation intensity (70–120% RMT) (Zhang et al., 2025), as well as differences in CBT modality (CBT, iCBT, and MBCT), treatment duration (2–8 weeks), and therapist background; (2) differences in patient clinical characteristics, including baseline depression severity, illness duration, prior treatment history, and comorbidity; (3) differences in assessment instruments, because although all studies used Hamilton depression scales, HAMD-17 and HAMD-24 differ somewhat in content and sensitivity; and (4) cultural background, as most included studies were conducted in Chinese populations (7 of 10), raising the possibility of culturally specific treatment response or reporting bias.

### 4.2 Additive effects versus synergistic effects

The terms additive effect and synergistic effect are often used interchangeably in the combined-intervention literature, although they refer to distinct conceptual and methodological constructs. An additive effect indicates that the combined intervention performs better than either component intervention alone. A synergistic effect, by contrast, implies a non-linear interaction such that the effect of the combined intervention exceeds the sum of the effects of the two component interventions. Demonstrating synergy requires more rigorous experimental designs, including single-treatment controls, sham or inactive controls, and adequate statistical power to test interaction effects (Tatti et al., 2022).

All 10 randomized controlled trials included in this review compared combined treatment with one or more single-treatment conditions and therefore provide supportive evidence for a possible additive effect of the combined intervention; in other words, the available evidence suggests that the combined approach may outperform single-treatment approaches in reducing depressive symptoms. Two four-arm trials (Li et al., 2023; Qiu et al., 2024) further strengthen this evidence base by showing the superiority of combined treatment relative to both CBT-based and rTMS-based single-treatment comparators within the same study populations.

By contrast, strict synergy cannot be directly established from the available data. Based on the two four-arm studies, we conducted a *post hoc* exploratory analysis by comparing the improvement associated with combined treatment with the summed improvements associated with the two component single interventions. In Li et al. (2023), the improvement in the combined-treatment group (8.71 points) did not exceed the sum of the single-treatment improvements (11.12 points). In Qiu et al. (2024), however, the improvement in the combined-treatment group (10.68 points) exceeded the summed improvement of the single interventions (5.72 points), suggesting a possible synergistic effect. These findings should be interpreted cautiously. Proper testing of synergy requires more rigorous methodological conditions, including sham stimulation controls, *a priori* power calculations, and prespecified interaction analyses. None of the included studies met these conditions, and synergy therefore remains a theoretical possibility rather than an empirically established finding. This conclusion is consistent with previous reviews, which have similarly suggested that the current evidence base primarily supports an additive framework, whereas claims of synergy remain largely theoretical and have yet to be rigorously tested in large randomized controlled trials (Khanna et al., 2026).

From a mechanistic perspective, the present findings are consistent with current frameworks proposing complementary effects of neuromodulation and psychotherapy. CBT acts primarily through top-down processing and targets maladaptive cognition and behavior via the dorsolateral prefrontal cortex (DLPFC), a region that often shows reduced functional activity in depression (Yang et al., 2018). rTMS may modulate prefrontal cortical excitability and related neural network activity while inducing neuroplastic changes, thereby creating a neural state that is more conducive to subsequent cognitive restructuring rather than acting solely through an independent antidepressant effect (Downar et al., 2024). Within this framework, the role of neuromodulation is to facilitate a neuroplastic state that supports psychotherapy. By promoting synaptic plasticity, rTMS may provide a more favorable neural substrate for subsequent cognitive-behavioral intervention, thereby increasing the likelihood that patients will benefit from psychotherapy. This interpretation is consistent with prior accounts emphasizing facilitation-based models of combined somatic and behavioral treatment (Gazerani, 2025).

The present findings support an additive interpretation of the combined intervention. That is, rTMS combined with CBT appears to provide greater clinical benefit than rTMS control, and qualitative evidence suggests possible advantages over medication-based CBT controls and medication controls. However, the available data do not establish a true synergistic effect. Demonstrating synergy would require designs capable of testing interaction effects, including adequate single-treatment controls, sham or inactive controls, sufficient statistical power, and prespecified interaction analyses. Therefore, claims of synergy should remain cautious until larger and more rigorous trials are available.

### 4.3 Strengths and limitations

This study has several strengths. First, it followed PRISMA guidelines and implemented a systematic search and screening process focused on randomized controlled trials, thereby reducing selection bias. Second, it included both English- and Chinese-language studies, increasing the breadth and representativeness of the evidence base and addressing a limitation of prior reviews dominated by English-language literature. Third, it synthesized the currently available randomized evidence and combined random-effects meta-analysis with sensitivity analysis and cumulative meta-analysis, thereby improving the robustness of the findings. Fourth, it focused specifically on the combined use of rTMS and CBT, providing a more targeted conclusion than prior reviews that included multiple psychotherapies or multiple brain stimulation techniques.

Several limitations should also be acknowledged. First, the number of included studies was limited (10 studies in total), and most were conducted in Chinese populations (7 of 10), which may reduce cross-cultural generalizability. Second, blinding was difficult to implement because of the nature of the interventions; the physical sensations associated with rTMS and the conversational nature of CBT meant that most studies were judged to have moderate-to-high risk of performance bias, which may have inflated effect estimates to some extent. Third, adverse events, cognitive or functional outcomes, and dropout or discontinuation were inconsistently reported across studies, which limited quantitative synthesis and the interpretation of safety and functional outcomes. Fourth, long-term follow-up data were sparse, as most studies reported only immediate post-treatment outcomes, preventing robust conclusions about the durability of benefit or relapse prevention. Only a few studies reported follow-up outcomes, and relapse was explicitly reported in only one study. Fifth, baseline medication use could not be fully controlled. Many studies allowed participants to continue existing antidepressant treatment during the trial, introducing potential confounding. In addition, the included trials were not designed to formally test interaction effects. Accordingly, the current evidence supports an additive interpretation of combined rTMS and CBT, whereas a genuine synergistic effect could not be verified. Although Egger’s test did not detect significant publication bias, the small number of included studies limits the sensitivity of publication-bias tests, and potential small-study effects cannot be ruled out.

### 4.4 Clinical and practical implications

This study provides systematic evidence supporting the clinical use of rTMS combined with CBT for depression. Meta-analytic findings indicate that the combined intervention yields greater improvement in depressive symptoms than rTMS control, while qualitative evidence suggests that it may also have advantages over medication-based CBT control and medication control. This integrated strategy, which combines neuromodulation with psychotherapy, appears to offer therapeutic benefit and clinical promise, particularly for patients who respond poorly to conventional pharmacotherapy or derive insufficient benefit from a single intervention.

At the same time, rTMS combined with internet-based CBT did not show a consistently enhanced effect. Its efficacy may be influenced by factors such as therapist guidance, cultural adaptation, and study quality. Accordingly, caution is warranted when considering iCBT as the psychotherapeutic component of combined treatment in clinical settings. Overall, the present findings support the complementary use of neuromodulation and cognitive-behavioral intervention and provide a basis for further standardization and clinical investigation of rTMS combined with CBT.

### 4.5 Implications for future research

Future research should advance this field in several directions. First, higher-quality study designs are needed, including multicenter, large-sample, prospective randomized controlled trials with improved blinding procedures (e.g., sham-rTMS controls and blinded independent outcome assessors) and longer follow-up periods (≥6 months) to evaluate maintenance of benefit and relapse prevention. Validation in non-Chinese populations is also needed to improve cross-cultural generalizability.

Second, treatment protocols should be standardized and optimized. Future research should standardize the reporting of key rTMS parameters, including stimulation site, frequency, intensity, pulse number, and treatment duration. It should also standardize CBT delivery characteristics, including modality, treatment length, frequency, and therapist qualifications. Dose–response studies are needed to identify the optimal “dose” of combined treatment. Given the opposing neurophysiological effects of high-frequency versus low-frequency rTMS, future studies should be designed with frequency stratification to allow direct comparisons between these two protocols and to clarify whether they exert differential moderating effects on the therapeutic efficacy of combination therapy.

Third, more precise subgroup analyses are needed. Future work should pay particular attention to adolescents and older adults and should clarify the neurobiological mechanisms through which age may influence treatment efficacy, potentially using neuroimaging, electrophysiological methods, or biological markers. It will also be important to examine differential treatment effects across depression subtypes (e.g., treatment-resistant depression, depression with anxious features, depression with cognitive impairment), illness course (first episode vs. recurrent depression), and comorbidity profiles to move toward more individualized care.

Fourth, mechanistic studies should be expanded. Combining functional magnetic resonance imaging, electroencephalography, and TMS–EEG methods may help clarify the neuroplastic mechanisms underlying the combined effects of rTMS and CBT. In particular, future work should examine whether rTMS modulates activity within networks such as the default mode network and executive control network in ways that facilitate the cognitive restructuring processes targeted by CBT. Clarifying these mechanisms may ultimately support more precise target selection and better-optimized treatment protocols.

Fifth, safety, cognitive, and discontinuation outcomes should be systematically reported in future trials. In the current evidence base, adverse events were generally mild and no serious adverse events were reported, but several studies did not report safety data at all. Cognitive outcomes were assessed in only a minority of studies, using heterogeneous instruments (e.g., RBANS, memory tests, MoCA, psychosocial functioning scales). Dropout and missing-data handling were also inconsistently reported. Future randomized controlled trials should prespecify and systematically collect these secondary outcomes using standardized instruments to enable quantitative synthesis and to provide a more comprehensive evidence base for clinical decision-making.

## 5 Conclusion

The rTMS combined with CBT appears to offer additional benefit in the treatment of depression. Meta-analytic evidence indicates that combined treatment produces greater improvement in depressive symptoms than rTMS control, while qualitative evidence suggests possible advantages over medication-based CBT control and medication control. The current evidence supports an additive interpretation of combining neuromodulation with CBT, but does not establish a true synergistic effect. Overall, the present study provides systematic support for the clinical value of rTMS combined with CBT in depression and offers a useful reference point for the design and optimization of future high-quality randomized controlled trials.

## Data Availability

The original contributions presented in the study are included in the article/Supplementary material, further inquiries can be directed to the corresponding author.

## Footnotes

1
https://osf.io/preprints/psyarxiv
