# Supplementary material for: Efficacy of repetitive transcranial magnetic stimulation combined with cognitive behavioral therapy for depression: a systematic review and meta-analysis
Source: Front Psychol. 2026 Jul 14;17:1867678. doi: 10.3389/fpsyg.2026.1867678 (PMC13408205; doi:10.3389/fpsyg.2026.1867678)
Supplement: Supplementary file 1 [file Data_Sheet_1.PDF]

Supplementary 1. Literature Search Strategy

We systematically searched seven databases, including Web of Science, PubMed, Embase, EBSCOhost, China National Knowledge Infrastructure (CNKI), Wanfang Data and VIP Database, for all relevant publications from their inception to December 1, 2025. A supplementary search was also conducted on PsyArXiv. No records retrieved from PsyArXiv met the inclusion criteria, and thus none were included in the final review and analysis. The search keywords for each database are presented below.

(1) Web of Science

(TS=("transcranial magnetic stimulat*" OR TMS OR rTMS OR "repetitive TMS" OR "brain stimulat*"))AND(TS=("cognitive behavioral therap*" OR CBT OR "cognitive therap*" OR "behavioral activation"))AND(TS=(depress* OR "major depressive disorder" OR MDD OR "treatment resistant depress*"))

(2) PubMed

("transcranial magnetic stimulat*"[tw] OR TMS[tw] OR rTMS[tw] OR "repetitive TMS"[tw] OR "brain stimulat*"[tw])AND("cognitive behavioral therap*"[tw] OR CBT[tw] OR "cognitive therap*"[tw] OR "behavioral activation"[tw])AND(depress*[tw] OR "major depressive disorder"[tw] OR MDD[tw] OR "treatment resistant depress*"[tw])

(3) Embase

#1 'transcranial magnetic stimulation'/exp OR 'transcranial magnetic stimulat*':ab,ti OR 'tms':ab,ti OR 'repetitive transcranial magnetic stimulation'/exp OR 'rtms':ab,ti OR 'repetitive tms':ab,ti OR 'brain stimulat*':ab,ti

#2 'cognitive behavioral therapy'/exp OR 'cognitive behavioral therap*':ab,ti OR 'cbt':ab,ti OR 'cognitive therapy'/exp OR 'cognitive therap*':ab,ti OR 'behavioral activation'/exp OR 'behavioral activation':ab,ti

#3 'depression'/exp OR 'depress*':ab,ti OR 'major depression'/exp OR 'major depressive disorder':ab,ti OR 'mdd':ab,ti OR 'treatment resistant depression'/exp OR 'treatment resistant depress*':ab,ti OR 'therapy resistant depression'/exp

#4 #1 AND #2 AND #3

(4) EBSCOhost

("transcranial magnetic stimulation" OR TMS OR rTMS OR "repetitive TMS" OR "brain stimulation") AND ("cognitive behavioral therapy" OR CBT OR "cognitive therapy" OR "behavioral activation") AND (depression OR "major depressive disorder" OR MDD OR "treatment resistant depression")

(5)CNKI (Chinese search terms were adopted for the Chinese databases)

检索式 SU=('经颅磁刺激' + 'TMS' + '重复经颅磁刺激' + 'rTMS' + '高频经颅磁刺激' + '低频经颅磁刺激') *('认知疗法' + '认知行为疗法' + '认知行为治疗' + 'CBT' + '认知治疗') * ('抑郁症' + '抑郁' + '抑郁发作' + '抑郁状态' + '抑郁症状')

(6) Wanfang Data (Chinese search terms were adopted for the Chinese databases)

检索式：(主题:(经颅磁刺激) OR 主题:(TMS) OR 主题:(重复经颅磁刺激) OR 主题:(rTMS) OR 主题:(高频经颅磁刺激) OR 主题:(低频经颅磁刺激)) AND(主题:(认知疗法) OR 主题:(认知行为疗法) OR 主题:(认知行为治疗) OR 主

题:(CBT) OR 主题:(认知治疗)) AND(主题:(抑郁症) OR 主题:(抑郁) OR 主题:(抑郁发作) OR 主题:(抑郁状态) OR 主题:(抑郁症状))
(7) VIP Database (Chinese search terms were adopted for the Chinese databases)
检索式：(M=经颅磁刺激 OR M=TMS OR M=重复经颅磁刺激 OR M=rTMS OR M=高频经颅磁刺激 OR M=低频经颅磁刺激) AND(M=认知疗法 OR M=认知行为疗法 OR M=认知行为治疗 OR M=CBT OR M=认知治疗) AND(M=抑郁症 OR M=抑郁 OR M=抑郁发作 OR M=抑郁状态 OR M=抑郁症状)
(8) PsyArXiv
TMS CBT depression

Supplementary 2. Exploratory Subgroup and Sensitivity Analyses

Exploratory subgroup analyses were conducted by CBT modality, rTMS stimulation frequency, and medication status. These analyses were used to examine potential sources of heterogeneity in the comparison between combined treatment and rTMS control. Because the number of studies in each subgroup was small, all subgroup findings should be interpreted cautiously.

For the subgroup analysis by CBT modality, studies were classified into face-to-face CBT and iCBT subgroups. The face-to-face CBT subgroup showed a significant benefit relative to rTMS control (Hedges' g = 0.51, 95% CI 0.24 to 0.79, I^2 = 54.8%), whereas the iCBT subgroup did not show a significant effect (Hedges' g = 0.04, 95% CI −0.33 to 0.41, I^2 = 0%). The between-subgroup difference was statistically significant (p = 0.041).

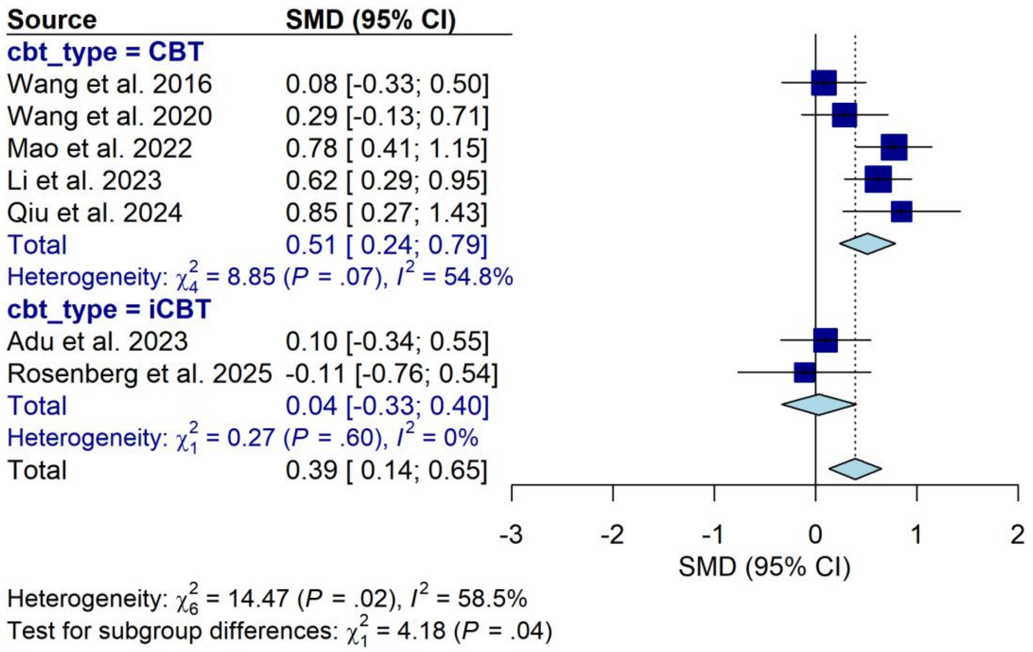

Figure R1. Subgroup analysis by CBT modality

For the subgroup analysis by rTMS stimulation frequency, studies were classified into

low-frequency, high-frequency, other-frequency, and mixed-frequency subgroups. The subgroup effects were as follows: low-frequency stimulation, Hedges' g = 0.37 (95% CI −0.16 to 0.89, I^2 = 74.6%); high-frequency stimulation, Hedges' g = 0.54 (95% CI −0.01 to 1.08, I^2 = 57.4%); other-frequency stimulation, Hedges' g = 0.38 (95% CI −0.49 to 1.24, I^2 = 81.3%); and mixed-frequency stimulation, Hedges' g = 0.10 (95% CI −0.34 to 0.55). The between-subgroup difference was not statistically significant (p = 0.668).

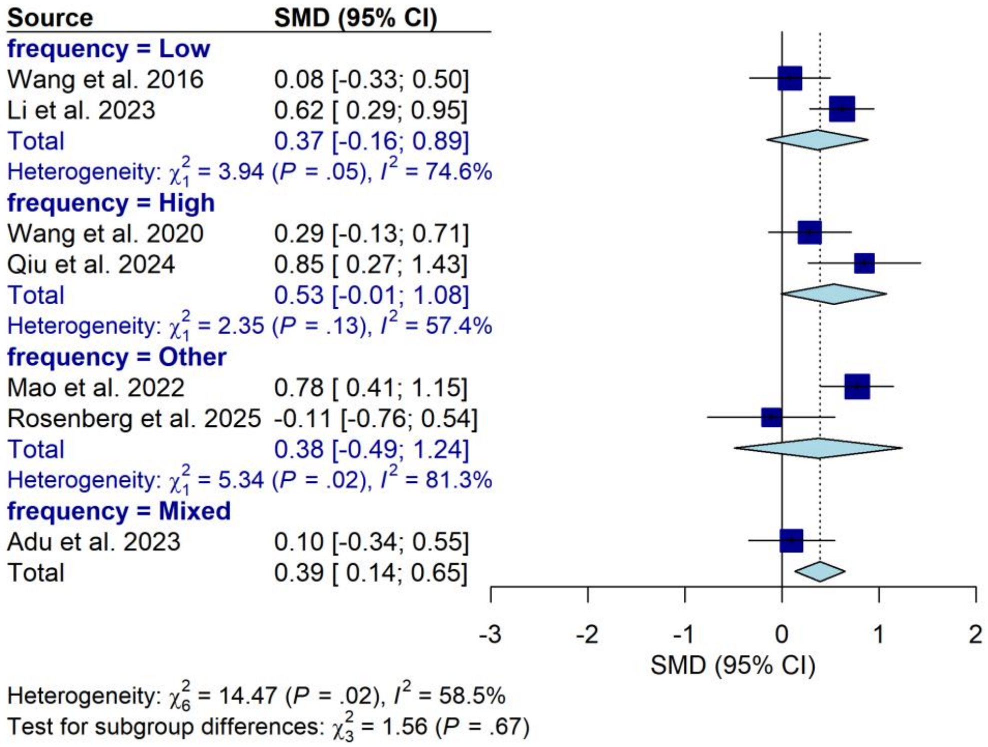

Figure R2. Subgroup analysis by rTMS stimulation frequency

For the subgroup analysis by medication status, studies were classified into concomitant medication and unreported medication-status subgroups. The concomitant medication subgroup showed Hedges' g = 0.50 (95% CI 0.07 to 0.93, I^2 = 65.5%), whereas studies with unreported medication status showed Hedges' g = 0.31 (95% CI −0.06 to 0.69, I^2 = 63.5%). The between-subgroup difference was not statistically significant (p = 0.523). Because "unreported" does not indicate "no medication," this analysis should be interpreted cautiously.

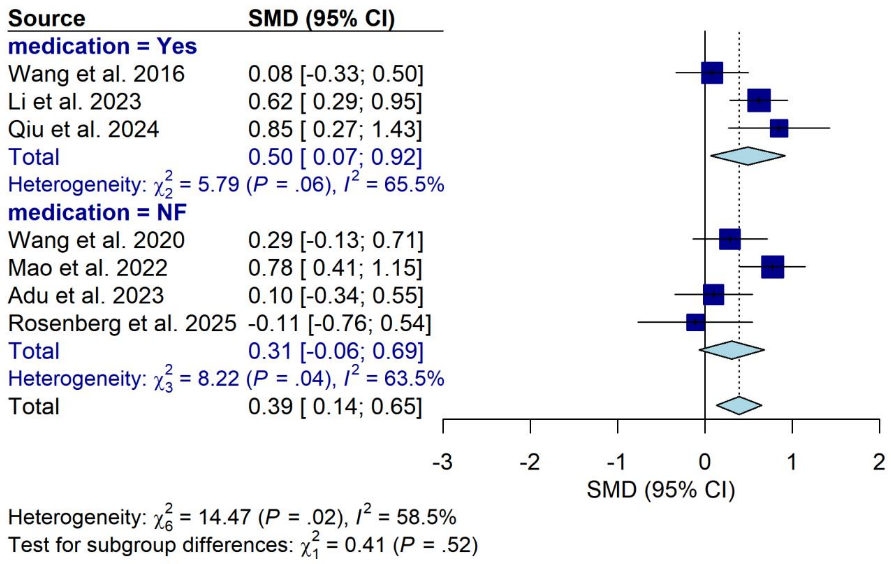

Figure R3. Subgroup analysis by medication status

To examine the robustness of the pooled estimate under different analytical assumptions, we conducted a sensitivity analysis using alternative assumed pre–post correlation coefficients. Because pre–post correlations were not reported in the original studies, the primary analysis assumed r = 0.5. We repeated the analysis using assumed correlations from 0.2 to 0.8 in increments of 0.1. Across all assumed values, the pooled effect remained positive and statistically significant. The pooled Hedges' g ranged from 0.34 to 0.50, and no confidence interval crossed zero, indicating that the estimated benefit of rTMS combined with CBT relative to rTMS control was robust to alternative assumptions regarding the pre–post correlation.

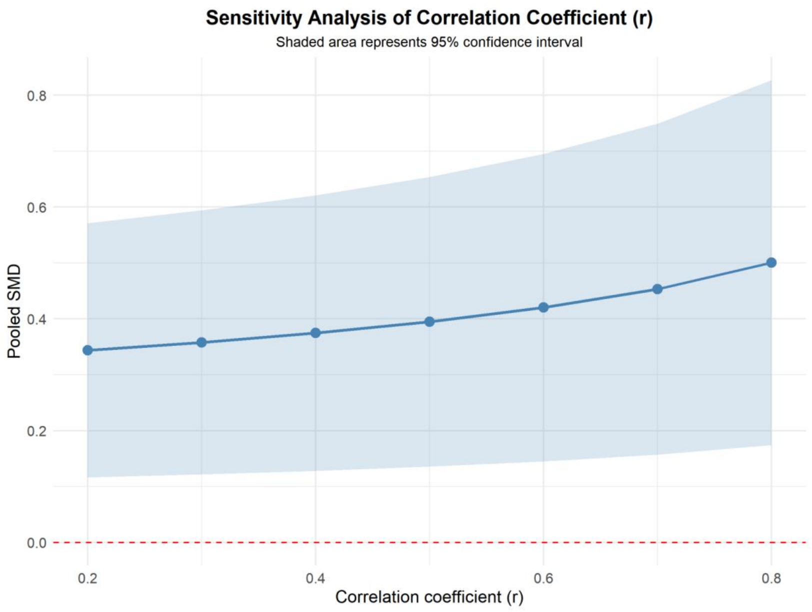

Figure R4. Sensitivity analysis using alternative assumed pre–post correlation coefficients

Supplementary 3. Operational Parameters of CBT-Spectrum Interventions

Operational parameters of CBT-spectrum interventions were extracted where available, including CBT modality, session duration, session frequency, treatment duration, total number of sessions, therapist involvement, and reporting notes. Because these parameters were incompletely reported across studies, they were summarized descriptively.

Table S1. Operational parameters of CBT-spectrum interventions in the included studies

Study	CBT modality	Session duration	Session frequency	Treatment duration	Total number of sessions	Therapist involvement	Main intervention content and reporting notes
Wang et al. (2016)	Traditional face-to-face CBT	Not reported	Not reported	4 weeks	Not reported	Face-to-face, therapist-guided	The CBT program was divided into four thematic stages: relationship building and cognitive assessment, cognitive restructuring, relaxation training, and behavioral modification/consolidation. Session duration and total number of sessions were not reported.
Wang et al. (2020)	Traditional face-to-face CBT	Not reported	Not reported	Not reported	Not reported	Face-to-face, therapist-guided	The study described CBT content, including psychological support and cognitive correction, but did not report session duration, session frequency, treatment duration, or total number of sessions.
Qi et al. (2021)	MBCT	45 min	Twice weekly	8 weeks	16 sessions	Therapist-guided	The intervention included raisin exercise, mindfulness concepts, body scanning, mindful breathing, observation of the relation between events, perceptions, and feelings, acceptance of thoughts and emotions, and

							engagement in pleasant activities.
Mao et al. (2022)	Traditional face-to-face CBT	30–40 min	Twice weekly	2 weeks	4 sessions	Face-to-face, therapist-guided	A short-term CBT protocol was delivered for two consecutive weeks.
Adu et al. (2023)	iCBT	60 min	At least twice weekly	6 weeks	12 sessions	Unguided self-help with SMS reminders	The study used the standardized MoodGYM platform. Patients completed the program at home, with SMS reminders but no real-time therapist supervision. Adherence monitoring was limited.
Li et al. (2023)	Traditional face-to-face CBT	Approximately 45 min	Twice weekly	8 weeks	16 sessions	Face-to-face, therapist-guided	CBT was delivered regularly across the treatment period.
Qiu et al. (2024)	Traditional face-to-face CBT	60 min	Once weekly	8 weeks	8 sessions	Face-to-face, therapist-guided	CBT was delivered once weekly during the intervention period.
Yao et al. (2024)	Traditional face-to-face CBT	Not reported	Not reported	Not reported	Not reported	Face-to-face, therapist-guided	The study described CBT content, including psychological support and cognitive correction, but did not report session duration, session frequency, treatment duration, or total number of sessions.
Dalhuisen et al. (2024)	Traditional face-to-face CBT	Not reported	Not reported	Not reported	Not reported	Therapist-guided	The study included CBT as part of the treatment condition, but detailed operational parameters, including session duration, frequency, and total number of sessions, were not reported.
Rosenberg et al. (2025)	iCBT	30-min online group discussion	Weekly	Not fully reported	Not clearly reported	Semi-guided; led by a mental health coach	The study used the This Way Up platform. The intervention included self-learning materials plus weekly online group discussions led by a professional mental health coach.

Supplementary 4. Secondary Outcomes Reported in the Included Studies

Secondary outcomes were extracted where available, including adverse events, cognitive or functional outcomes, and dropout or discontinuation. Because these outcomes were inconsistently reported and assessed using heterogeneous measures, they were summarized narratively rather than pooled quantitatively.

Table S2. Secondary outcomes reported in the included studies

Study	Adverse events	Cognitive or functional outcomes	Dropout/discontinuation	Reporting notes
Wang et al. (2016)	Mild adverse reactions were reported in all three groups, including gastrointestinal symptoms (dry mouth, nausea, diarrhea), dizziness, somnolence, and sweating. All adverse reactions resolved spontaneously after approximately one week without special treatment, and no significant between-group differences were reported.	Cognitive and psychosocial functioning were indirectly reflected by the Tennessee Self-Concept Scale (TSCS) and Personal and Social Performance scale (PSP). The combined-treatment group showed higher TSCS scores (225.39 ± 16.43 vs. 207.19 ± 9.84) and higher PSP scores (75.98 ± 10.61 vs. 61.99 ± 5.87) than the control group.	Not clearly reported.	Relapse outcomes were reported during follow-up. Follow-up assessments were conducted at 1, 3, and 6 months after treatment. At 6 months, three relapses (6.67%) occurred in the sertraline-only group, whereas no relapse was observed in the medication plus low-frequency rTMS group or in the medication plus rTMS combined with CBT group.
Wang et al. (2020)	Not reported.	Not reported.	Not clearly reported.	Secondary outcomes were not sufficiently described.
Qi et al. (2021)	Not reported.	RBANS was used. The combined-treatment group showed a higher total RBANS score after intervention than the control group (305.44 ± 8.37 vs. 275.90 ± 15.16, $p < 0.05$). The combined-treatment group also scored higher in attention (73.35 ± 6.57 vs. 68.05 ± 8.22),	Not clearly reported.	Cognitive outcomes were reported, but safety and dropout data were incomplete.

		language (64.93 ± 4.52 vs. 57.60 ± 6.01), visuospatial/constructional ability (72.30 ± 4.66 vs. 65.72 ± 4.83), immediate memory (47.94 ± 4.32 vs. 42.65 ± 5.68), and delayed memory (46.92 ± 5.45 vs. 41.88 ± 5.31).		
Mao et al. (2022)	Not reported.	The Wechsler Memory Scale was used. Lower scores indicated better memory function. The combined-treatment group showed lower scores than the control group in recognition (8.32 ± 2.09 vs. 8.94 ± 2.11), picture memory (5.38 ± 1.31 vs. 6.89 ± 1.65), associative memory (6.89 ± 2.11 vs. 7.48 ± 2.16), and digit span (6.44 ± 1.46 vs. 7.42 ± 1.61).	Not clearly reported.	Memory outcomes were reported, but adverse events and dropout were not clearly described.
Adu et al. (2023)	The most common rTMS-related side effects were transient headache, dizziness, and scalp discomfort at the stimulation site. No serious adverse events occurred.	Not reported.	An overall dropout rate of approximately 28% was reported. Reasons for refusal or non-participation included lack of interest in the study and time constraints.	iCBT adherence and attrition were relevant concerns.
Li et al. (2023)	The study noted that there was no significant difference in the incidence of adverse events among the four groups, but specific event types, case numbers, and incidence rates were not reported.	MoCA was used as a baseline inclusion criterion (⩾25) but was not assessed as a pre-post cognitive outcome.	Attrition was reported. Overall dropout rate was 6.4% (20/312).	Safety and dropout data were reported, but details were insufficient for quantitative synthesis.
Qiu et al. (2024)	Not reported.	Not reported.	Not clearly reported.	Secondary outcomes were not sufficiently described.
Yao et al. (2024)	Not reported.	MoCA was used. The combined-treatment group had a higher post-treatment MoCA	Not clearly reported.	Cognitive outcomes were reported, but adverse events and

		score than the control group (27.76 ± 2.95 vs. 26.86 ± 3.17, p = 0.012).		dropout were not clearly described.
Dalhuisen et al. (2024)	Not clearly reported.	Quality-adjusted life years (QALYs) were reported as part of the health-economic evaluation.	Attrition was reported. Only 44.9% of patients completed data collection at all time points, indicating substantial missing data.	Follow-up and health-economic outcomes were reported, but adverse-event reporting was limited.
Rosenberg et al. (2025)	No deaths or serious adverse events were reported in either group.	Not reported.	Follow-up data were incomplete because of the small sample size and high dropout rate.	Planned 6- and 9-month follow-ups were included, but the available data did not support reliable long-term efficacy or relapse analysis.
